# Serine Proteases of Malaria Parasite *Plasmodium falciparum*: Potential as Antimalarial Drug Targets

**DOI:** 10.1155/2014/453186

**Published:** 2014-03-11

**Authors:** Asrar Alam

**Affiliations:** Department of Biological Sciences, Tata Institute of Fundamental Research, Homi Bhabha Road, Colaba, Mumbai 400005, India

## Abstract

Malaria is a major global parasitic disease and a cause of enormous mortality and morbidity. Widespread drug resistance against currently available antimalarials warrants the identification of novel drug targets and development of new drugs. Malarial proteases are a group of molecules that serve as potential drug targets because of their essentiality for parasite life cycle stages and feasibility of designing specific inhibitors against them. Proteases belonging to various mechanistic classes are found in *P. falciparum*, of which serine proteases are of particular interest due to their involvement in parasite-specific processes of egress and invasion. In *P. falciparum*, a number of serine proteases belonging to chymotrypsin, subtilisin, and rhomboid clans are found. This review focuses on the potential of *P. falciparum* serine proteases as antimalarial drug targets.

## 1. Global Malaria Burden and Need for Development of Novel Antimalarials

Malaria caused by protozoan parasite* Plasmodium* is a major global parasitic disease [1]. Malaria in humans is caused by five* Plasmodium* species, namely,* P. falciparum*,* P. vivax*,* P. ovale*,* P. malariae*, and* P. knowlesi*. Of these,* P. falciparum* is the causative agent of severe malaria and the major cause of malaria-related fatality.

According to World Malaria Report of 2013, there were an estimated 207 million clinical cases of malaria in 2012 and an estimated 627,000 deaths, with about 90% of deaths occurring in sub-Saharan Africa. International efforts to control malaria have resulted in significant reduction of malaria-related deaths. Between 2000 and 2012, malaria-related deaths reduced by 29% globally and 31% in the WHO African Region [2]. Methods used to prevent the spread of the disease or to protect individuals in areas where malaria is endemic include therapeutic and prophylactic drugs, mosquito eradication, and prevention of mosquito bites by using insecticide-treated nets (ITNs), indoor residual spray, and larval control [2].

Early antimalarial agents were isolated from natural products. Bark of the cinchona tree and extracts of the wormwood plant were among the first effective antimalarials. Quinoline compound chloroquine has been the most widely used drug until recently. Resistance to chloroquine started in Africa in the 1980s, causing tremendous resurgence of malaria burden [3, 4]. Chloroquine resistance prompted many countries to adopt sulfadoxine-pyrimethamine (SP) as the first-line antimalarial but resistant* P. falciparum* populations were selected quickly in Africa, Southeast Asia, and South America. It was abandoned after only 5 years of use in Southeast Asia [5, 6]. Due to widespread resistance to the available antimalarials, artemisinin-based combination therapies (ACTs) were introduced in Asia, Africa, and South America. The artemisinins are potent and rapidly acting antimalarials derived from the Chinese sweet wormwood plant,* Artemisia annua* [7, 8]. Due to their short duration of action, artemisinins cannot be administered alone, which results in recrudescent parasitemia [9]; however, they can be administered as ACTs over three days in the combinations with longer-acting antimalarials in the forms of artemether-lumefantrine, amodiaquine-artesunate, and mefloquine-artesunate [10]. Despite the effectiveness of ACTs, use of artemisinin monotherapy resulted in emergence of drug-resistant* P. falciparum* parasites in Cambodia-Thailand border region [11, 12]. According to WHO, till now drug resistance has been reported in three* Plasmodium* species,* P. falciparum*,* P. vivax*, and* P. malariae* [13].

Currently treatment of malaria is effected mainly through the administration of chloroquine, SP, and ACTs. Prophylactic drugs include chloroquine, primaquine, mefloquine, doxycycline, and malarone (atovaquone and proguanil) [14]. Despite the availability of antimalarials for both treatment and prophylaxis, the spread of resistance and paucity of more antimalarials warrants the need for identification of new drug targets and development of novel drugs.

## 2. Proteases as Antimalarial Drug Targets

Proteases constitute a ubiquitous and highly abundant group of catalytic and regulatory molecules having widespread roles in living systems. They are primarily involved in protein turnover to their constituent amino acids to generate the building blocks for new proteins and digestion of dietary proteins in higher organisms. Besides, protein activation by limited proteolysis is a common means of regulation of many physiological processes [15]. Proteases constitute the major virulence factors in various parasitic diseases such as schistosomiasis, malaria, leishmaniasis, Chagas disease, and African sleeping sickness. Some well-characterized examples of the roles of proteases in parasite pathogenesis include their involvement in the invasion of host cells, degradation of hemoglobin and other blood proteins, immune evasion, and activation of inflammation [16]. In this context, they are crucial for the pathogenic organisms both for their survival and the diseases they cause. Their potential as drug targets is underscored by the feasibility of designing specific inhibitors against them.

Proteases recognize an optimum peptide sequence and catalyze its cleavage at the active site. Selective inhibitors targeting the active sites can be developed. Besides the active sites, exosites and allosteric sites also participate in substrate recognition. Hence, selective inhibitors targeting these sites can also be developed [17].

Protease inhibitors have been successfully used as drugs against human immunodeficiency virus (HIV) [18] and hepatitis C virus (HCV) [19] and in treatment of hypertension [20] and coagulopathies [21]. The active sites of proteases have been successfully targeted against viruses HIV and HCV and angiotensin-converting enzyme in hypertension [22, 23]. Targeting the active site is not always feasible due to homology with the host enzymes. For example, in many cancers, development of protease inhibitor-based drugs has been challenging due to the difficulty in selectively targeting the active sites. In such cases, allosteric sites could be targeted to achieve the goals [17]. Malaria parasite is the most important member of the parasites of phylum Apicomplexa, which invade the host cell and reside in intracellular niche that is protected from host defenses and provides a rich source of nutrient. Asexual erythrocytic life cycle of malaria parasite is responsible for the clinical symptoms of malaria. It starts with the invasion of erythrocytes by the merozoites released from liver. The intraerythrocytic parasite feeds on host hemoglobin and develops from small ring stage form to a relatively large and metabolically active trophozoite stage parasite, which then transforms to multinucleated schizont. Inhibitor-based studies have shown that cysteine, aspartic, metallo, and serine protease activities are crucial for completion of this cycle [24]. Several previous studies have implicated a functional role of serine proteases in egress and invasion at blood stages [25–27].

## 3. *P. falciparum* Serine Proteases 

Serine proteases are widely dispersed in organisms through evolution and have diverse functions. They have been grouped into thirteen clans [28]. Chymotrypsin/trypsin-like and subtilisin-like serine proteases are two major clans of serine proteases, which have highly similar arrangement of catalytic triad Asp, His, and Ser residues and radically different protein scaffolds, that is, *β*/*β* for chymotrypsin and *α*/*β* for subtilisin [29]. A number of serine proteases belonging to chymotrypsin, subtilisin, and rhomboid protease clans are found in* P. falciparum* genome. A list of* P. falciparum* serine proteases along with their orthologs and putative functions is presented in Table S1 (Supplementary Materials available online at http://dx.doi.org/10.1155/2014/453186). These proteases are expressed in a temporally regulated manner at the asexual and sexual stages of the parasite life cycle [30–32]. Table S2 presents the microarray and mass spectrometry based expression profiles of* P. falciparum* serine proteases. Some of these proteases are known to be essential for parasite development at the erythrocytic and exoerythrocytic stages suggesting their potential as targets for therapeutic intervention.

### 3.1. *P. falciparum* Chymotrypsin-Like Proteases

Two genes encoding for serine proteases of chymotrypsin-like clan (PlasmoDB IDs: PF3D7_0807700 and PF3D7_0812200) were identified in* P. falciparum* genome. PF3D7_0807700 is homologous to DegP heat shock protein family [33]. Since DegP acts as a chaperone at low temperature and protease at elevated temperature, its role in extracellular process related to invasion is unlikely. The second putative chymotrypsin-like serine protease PF3D7_0812200 possesses PDZ2 domain besides the trypsin domain. This domain is found in prokaryotic, viral, and eukaryotic signaling proteins having GTPase activity [34], known to anchor transmembrane proteins to cytoskeleton and assembly of signaling complexes. This protease is also unlikely to be directly involved in invasion.

### 3.2. *P. falciparum* Subtilisin-Like Proteases

Three genes encoding for proteases of another major clan, subtilisin-like proteases or subtilases (clan SB) [35], are found in* P. falciparum* genome known as PfSUB1, 2, and 3. All of them are highly expressed at late asexual blood stages [31]. Of these, PfSUB1 and 2 have been extensively characterized and implicated in egress and invasion during asexual blood stage life cycle of the parasite [26, 27, 36]. PfSUB3 is the least characterized member and preliminary reports have confirmed the* in vitro* serine protease activity of PfSUB3 and also identified a multifunctional parasite protein, profilin, as its interacting partner [37, 38].

#### 3.2.1. *P. falciparum* Subtilisin-Like Protease 1 (PfSUB1)

PfSUB1 (PlasmoDB ID: PF3D7_050700 and MEROPS identification number S08.012) is the first identified member of* P. falciparum* subtilases. The primary structure of PfSUB1 classifies it in a small group of bacterial-like eukaryotic subtilases [29, 35]. PfSUB1 undergoes two major intracellular processing steps during maturation. The first one takes place inside the lumen of endoplasmic reticulum and converts the earliest detectable 82 kDa form into a 54 kDa form (p54) [39]. The second, brefeldin A-sensitive processing step, is the conversion of p54 to intracellular 47 kDa terminal processing product (p47); both p54 and p47 contain the predicted catalytic domain [40].

Expression of codon-optimized PfSUB1 gene in recombinant baculovirus-infected insect cells resulted in the secretion of the processed form (p54) [39]. N-terminal radiosequencing of the* in vitro* translated protein showed the cleavage between Asp^219^ and Asn^220^ within the sequence Leu-Val-Ser-Ala-Asp-Asn-Ile-Asp-Ile-Ser. This highly restricted substrate specificity of PfSUB1 is suggestive of a very specialized and nondegradative biological function in the parasite [39, 41]. A substantial fraction of insect cell-secreted p54 was found bound to its 31 kDa propeptide (rp31), which was a highly selective, high-affinity inhibitor of the protease with dissociation constant in nanomolar range (Ki~12.5 nM) [39]. Truncation of 11 residues from the C-terminal of rp31 substantially reduced inhibition of PfSUB1 activity [42]. Since subtilase propeptides are specific inhibitors of their cognate proteases [43–47], the inhibitory peptides from the proregion will facilitate designing of specific inhibitors of the protease.

Many* Plasmodium* proteins possess structural insertions not found in their homologs from other genera [48, 49]. These insertions may provide targets for highly selective therapies against* P. falciparum*. Comparison of PfSUB1 primary structure with its orthologs and related bacterial subtilisins revealed the presence of both high and low complexity insertions that are predicted to form surface strand or loop structures. Site-directed mutagenesis, deletion of the whole loop insertions, or strategic replacements revealed that the majority of the loop insertions are critical for the activity of the protease [50].

PfSUB1 gene was found to be refractory to deletion in blood stages. It was stored in apical organelles, distinct from those involved in erythrocyte invasion and termed as “exonemes” [26]. During the final stage of schizont maturation, it was discharged into the parasitophorous vacuole (PV) and triggered a series of proteolytic events resulting in merozoite egress [27]. In an attempt to identify the mediators of egress, Arastu-Kapur et al. tested the effect of a library of 1,200 focused serine and cysteine protease inhibitors on blood stage malaria parasite growth. Using the hits from library screening, they identified PfSUB1 and dipeptidyl aminopeptidase 3 (DPAP3) as the primary regulators of egress. Inhibition of PfSUB1 and DPAP3 caused a block in schizont rupture [27] whereas at a relatively lower concentration of the inhibitor, defective merozoites were released [51]. DPAP3 caused maturation of PfSUB1 [27]. The mature PfSUB1 caused processing of parasitophorous vacuolar proteins SERA5 [27] and SERA6 [52], which are implicated in merozoite egress.

Merozoite surface protein 1 (MSP1) complex is a large glycosylphosphatidylinositol (GPI)-anchored protein complex, which is comprised of MSP1 and its associated partner proteins MSP6 and MSP7. During erythrocyte invasion, the initial low affinity interaction with the host cell takes place through this complex [53–55]. Proteolytic processing (primary processing) of this complex is necessary for initial low affinity interaction between the host and parasite and erythrocyte invasion. At a later stage, another processing of this complex is required for movement of the merozoite inside the host cell (secondary processing) [36]. PfSUB1 carries out the primary processing of MSP1 complex in the parasitophorous vacuole in a spatiotemporally regulated manner [56]. Besides, PfSUB1 also cleaves a number of merozoite and parasitophorous vacuolar proteins [57]. Besides blood stages, SUB1 is also essential for liver developmental stages. Conditional knockout of* Plasmodium berghei* SUB1 (PbSUB1) revealed that SUB1, although not essential for early liver stage development, was essential for development of liver stage schizonts and production of merozoites [58]. Conditional mutagenesis studies showed that PbSUB1-deficient merozoites were unable to egress from the hepatocytes [59].

Recently attempts have been made to identify PfSUB1 inhibitors. Maslinic acid (MA), a low toxic natural pentacyclic triterpene, was found to inhibit the* P. falciparum* blood stage transition from ring to schizont stage by a multitargeted mechanism. MA was found to inhibit the proteolytic processing of the MSP1 complex, probably by targeting PfSUB1 [60]. Characterization of PfSUB1 orthologs from* P. vivax*,* P. knowlesi*, and* P. berghei* revealed that although there are a number of unusual features of the SUB1 substrate binding cleft, cleavage sites in parasite substrates in these proteases are conserved. Two peptidyl alpha-ketoamide inhibitors of PfSUB1 inhibited all its orthologs suggesting that small molecule inhibitors can be developed against this protease [61]. A molecular dynamics simulation study of binding of known PfSUB1 substrate peptides based on its prodomain revealed that the prime and nonprime sides of the scissile bond make the major contribution to the binding free energy. It comprises the peptide residues P4 to P2′ making this region of potential interest for designing peptidomimetic inhibitors against PfSUB1 [62]. Given its essentiality for parasite blood and liver stages, proteolytic activity on multiple parasite proteins, and role in egress and invasion, PfSUB1 qualifies as an attractive antimalarial drug target.

#### 3.2.2. *P. falciparum* Subtilisin-Like Protease 2

*P. falciparum* subtilisin-like protease 2 (PfSUB2) (PlasmoDB ID: 248 PF3D7_1136900 and MEROPS identification number: S08.013) is a type I 249 transmembrane protein and expressed at late asexual blood stages. Attempts to disrupt* P. berghei* ortholog of PfSUB2 (PbSUB2) by double-crossover integration have been unsuccessful, suggesting the potential of PfSUB2 as a drug target [63]. It is secreted into merozoite apical organelles “micronemes” and plays a critical role in merozoite invasion of red blood cells (RBCs) [36].

PfSUB2 causes shedding of merozoite adhesins MSP1 and apical membrane antigen 1 (AMA1) at a juxtamembrane site during invasion [64]. Cleavage of MSP1 by PfSUB2 takes place distal to an epidermal growth factor- (EGF-) like domain at its C-terminal called MSP1_19_ [53]. MSP1_19_ remains bound to the merozoite surface and it is the only part of MSP1, which enters into the host cell. Cleavage of AMA1 takes places 29 residues away from the transmembrane domain, releasing the bulk of the ectodomain. In this way, the juxtamembrane “stub” along with its cognate transmembrane domain (TMD) and cytoplasmic domain enters into the host cell [65]. Shedding of these proteins is essential for productive invasion [65–67]. Since PfSUB2 causes the shedding of both MSP1 and AMA1 at the moving junction during erythrocyte invasion, it is termed as “merozoite surface sheddase” (MESH). This protein has not been expressed in recombinant proteolytically active form but shows the MESH activity in purified merozoites. It translocates from the anterior to the posterior end of the merozoite in an actin-dependent movement as the merozoite enters into the host erythrocyte [36].

Like PfSUB1, PfSUB2 also undergoes proteolytic processings in the parasite, which could be probable maturation events. The open reading frame encoding for PfSUB2 was* in vitro* translated, which revealed that the 154.8 kDa primary translated product (SUB2p) underwent rapid conversion to 74 kDa intermediate species (SUB2_I_) which was quantitatively converted to terminal 72 kDa species (SUB2_T_) [68]. The prodomain of PfSUB2 has been found to be a selective inhibitor of its “sheddase” activity [36]. Nuclear magnetic resonance (NMR) structure of PfSUB2 prodomain identified a likely catalytic domain-binding interface region in it, which could be exploited to design peptidomimetic inhibitor against the protease [69]. Essentiality of the protease for parasite survival, involvement in RBC invasion, and the initial findings suggesting the feasibility of designing inhibitors against the protease make PfSUB2 a promising drug target against malaria.

#### 3.2.3. *P. falciparum* Subtilisin-Like Protease 3

*P. falciparum* subtilisin-like protease 3 (PfSUB3) (PlasmoDB ID: PF3D7_0507200 and MEROPS identification number: S08.122) is the third* P. falciparum* subtilase. PfSUB3 is the least studied member of* P. falciparum* subtilases. It is also highly expressed at late asexual blood stages [31]. The full-length PfSUB3 gene encodes an 88 kDa protein, the 25 kDa C-terminal region of which has been shown to possess serine protease activity [37]. Yeast two-hybrid screening has revealed parasite profilin (PfPRF), a cytoskeletal and proinflammatory molecule, as an interacting partner of PfSUB3. PfPRF was found to induce the secretion of proinflammatory cytokines IL-12 and TNF-*α* from mouse bone marrow-derived dendritic cells. PfSUB3 showed proteolytic activity on PfPRF in* in vitro* assays and caused cleavage of PfPRF into multiple fragments of smaller sizes, which were hydrolyzed by increasing concentration of PfSUB3 [38]. It is still not clear if this proteolytic activity causes maturation of PfPRF or degradation under physiological conditions. Given the serine protease activity of PfSUB3 and multiple physiological functions of PfPRF, namely, motility, egress, and induction, of proinflammatory cytokines, its role in the related processes needs to be explored [38].

### 3.3. *P. falciparum* Rhomboid Proteases

Rhomboid proteins are intramembranous serine proteases with their catalytic triad embedded within the membrane bilayer, surrounded by a hydrophilic cavity formed by a protein ring [70]. Nine rhomboid protease genes are found in* P. falciparum* genome.* P. falciparum* rhomboids are largely uncharacterized till date.

Two characterized members of* P. falciparum* rhomboids are PfROM1 and PfPROM4. PfROM1 localizes to a thread-like apical organelle of blood stage merozoites termed as “mononeme” [71] and on the surface of sporozoites after salivary gland invasion [72].* Plasmodium yoelii* ROM1 deficient parasites were attenuated during erythrocytic and hepatic stages and defective in parasitophorous vacuole (PV) development [73]. PfROM1 and PfROM4 helped in merozoite invasion by catalyzing the intramembrane cleavage of the merozoite adhesin AMA1 [65, 74] and erythrocyte binding antigen 175 (EBA-175), respectively [75]. PfROM1 and/or 4 were able to cleave a variety of adhesins involved in host parasite interaction within the transmembrane domains [74]. Although initial reports on these proteases are suggestive of their importance for parasite development, they still remain to be extensively characterized and assessed for their therapeutic value.

## 4. Conclusion

**Figure 1:**
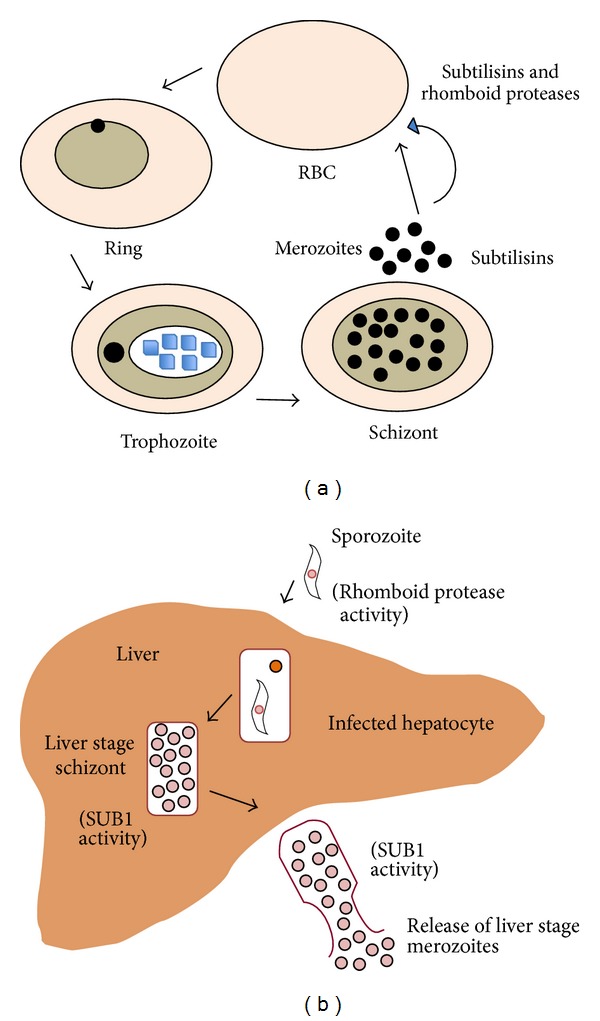
Role of serine proteases at asexual blood stages (a) and liver stages of* Plasmodium falciparum* (b). Subtilisin-like proteases are essential for merozoite invasion and egress in blood stages, liver stage schizont development and subsequent liver stage merozoite egress. Rhomboid protease activities are supposed to be involved in invasion of RBC and liver.

*P. falciparum* serine proteases are of particular interest as potential antimalarial drug targets due to their role in the processes of egress and invasion at erythrocytic and preerythrocytic stages, two critical checkpoints where the parasite development can be blocked. Involvement of parasite serine proteases in processing of parasite molecules involved in molecular interactions during parasite invasion and cleavage of the transmembrane adhesins for the invasion make them attractive drug targets. Liver stages, although clinically silent, are potential targets of drug and vaccine intervention due to their low abundance and distinct metabolism. Study of proteases expressed at liver stages is an exciting area of research. A schematic diagram of role of* P. falciparum* serine proteases at asexual blood stages and liver stages is shown in Figure 1.

Extensive biochemical and structural characterization of these molecules and high throughput screening for small molecules inhibitors will lead to the way of development of novel drugs directed against these proteases. Besides, the peptidomimetic inhibitors based on the inhibitory region of the prodomains can also be developed.

## Supplementary Material

Table S1. List of P. falciparum serine proteases of different classes with their functional domains Genes encoding for P. falciparum serine proteases of chymotrypsin, subtilisin and rhomboid protease clans and their orthologs found in PlasmoDB database (http://www.plasmodb.org/) are listed in the table. Domains present in these proteins were identified by sequence search against Pfam database (http://www.pfam.sanger.ac.uk/). Functions of these proteins if known from published literature are also listed.
Table S2. List of *P. falciparum* serine proteases with their expression at asexual and sexual stages of the parasite Highest expression of P. falciparum serine proteases at asexual blood stages as determined by microarray analysis and mass spectrometry-based evidence of expression at asexual and sexual stages as deposited in PlasmoDB database (http://www.plasmodb.org/) are presented. Ring, trophozoite and schizont represent asexual blood stage forms.
